# Promising Hybrids Derived from S-Allylcysteine and NSAIDs Fragments against Colorectal Cancer: Synthesis, *In-vitro* Evaluation, Drug-Likeness and *In-silico* ADME/tox Studies

**DOI:** 10.22037/ijpr.2020.114347.14806

**Published:** 2021

**Authors:** Angie Herrera-R, Wilson Castrillón, Manuel Pastrana, Andres F. Yepes, Wilson Cardona-G

**Affiliations:** *Química de Plantas Colombianas, Institute of Chemistry, Faculty of Exact and Natural Sciences, University of Antioquia (UdeA), Calle 70 No. 52–21, A.A 1226, Medellín, Colombia.*

**Keywords:** S-allyl cysteine, NSAIDs, Hybrid, Cell death, Colorectal cancer, In-silico, ADME

## Abstract

We synthesized twelve hybrids, *S*-allyl Cysteine methyl, ethyl and propyl ester-based non-steroidal anti-inflammatory drugs and their structures were elucidated by spectroscopic analysis. The chemopreventive potential of all compounds was evaluated against SW480 human colon adenocarcinoma cells and the non-malignant CHO-K1 cell line. Among the tested compounds, hybrids **10b-c**, **11b** and **12b** displayed the best anticancer activity with IC_50_ values between 0.131-0.183 mM and selectivity indices higher than 1 after 48 h of treatment. Selectivity indices were comparable to those reported for the reference drug, 5-fluorouracil (SI > 1). The SAR analysis showed that compounds with two carbon atom alkylic chains displayed the best activity (**10b**, **11b** and **12b**). Modeling studies including drug-likeness, bioactivity score and ADME/tox studies using online tools like molinspiration and Osiris suggested that these designed hybrids have a good pharmacological profile and can be considered as promising scaffolds for further studies in the search for new therapeutic alternatives to treat colorectal cancer.

## Introduction

Colorectal cancer (CRC) is one of the leading causes of morbidity and mortality worldwide (1). According to the most recent statistics, CRC has exhibited a significant increase through the years, being nowadays the second most lethal cancer, preceded by lung cancer. Current chemotherapy for CRC involves multi-drug treatments such as FOLFIRI (folic acid/5-FU/irinotecan), FOLFOX (5-FU/leucovorin/oxaliplatin) and FOLFOXIRI (leucovorin/5-FU/oxaliplatin/irinotecan) that are composed of 5-fluorouracil as the backbone of treatment. Although these therapeutic schemes are effective, they cause many adverse effects in the nervous and gastrointestinal systems, often associated with the cessation of anti-cancer therapy (2, 3). Because of that, it is necessary to discover new more potent and selective agents for treating this disease. 

*S*-allyl cysteine (SAC) is constituent of garlic, which has exhibited antioxidant properties both* in-vitro *(4) and *in-vivo* (5-7), antiproliferative effects on neuroblastoma (8) and melanoma (9), cell cycle arrest at the G0/G1 phases in prostate carcinoma cells and apoptosis with decreased in Bcl-2 expression and increased expression of Bax and caspase 8 in the same cells (10,11).

Non-steroidal anti-inflammatory drugs (NSAIDs) are a chemically heterogeneous group of compounds sharing certain therapeutic actions related to nociception, inflammation and pyretic processes. Among NSAIDs are included aspirin, diclofenac, naproxen and ibuprofen. The principal therapeutic effect of NSAIDs is the inhibition of cyclooxygenase (COX) family proteins which catalyze the rate-limiting steps in prostaglandin synthesis. Prostaglandins are important mediators of signal transduction pathways, and they are involved in cellular adhesion, growth and differentiation. In recent years, the possible role of NSAIDs in the prevention of malignancy has aroused interest (12-15). Several studies suggest that NSAIDs reduce the risk of and mortality from colon cancer by about half and constitute the prototypical colon cancer chemopreventive agents (16). Besides, these also inhibit the growth of other cell lines such as breast, lung and ovary cancer (17-22).

**Figure 1:**
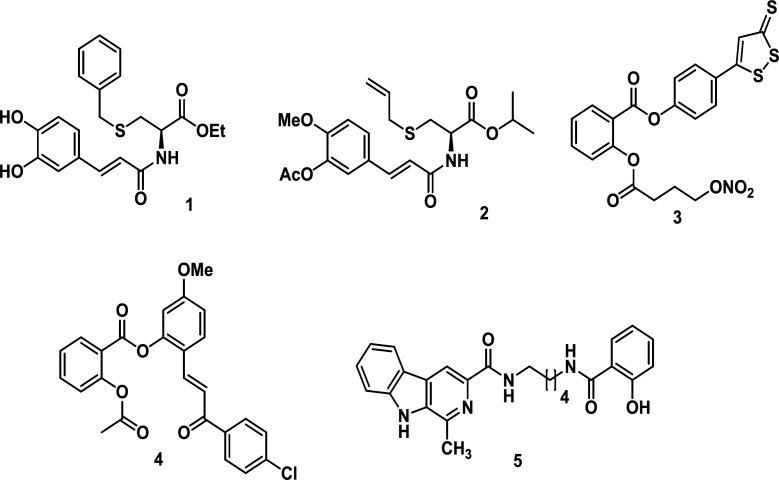
Hybrid molecules derived from *S*-allyl cysteine and salicylic acid with anticancer activity

Different studies have shown hybrid molecules as promising agents in medicinal chemistry and drug discovery research (23) since these compounds combine two or more structural fragments with relevant pharmacological and biological action (24, 25), suggesting they could display dual activity (26, 27). The caffeoyl amide of L-cysteine (**1**) exhibited good radical scavenging activity, being superior to the standards ferulic acid, eugenol and isoeugenol (28). SAC derivative (**2**) exhibited butyrylcholinesterase (BuChE) inhibitory activity (29). The hybrid, NOSH–aspirin (**3**), is an NO- and H_2_S-releasing agent. NOSH–aspirin inhibited cell growth, proliferation, induced apoptosis and caused G0/G1 cell cycle arrest in HT-29 colon cancer cells (30). Compound **4**, a hybrid molecule derived from aspirin and chalcone, inhibited proliferation of CRC cell lines with better activity regarding the reference drug -5-Fluorouracil-. Besides, it displayed 8-fold less inhibitory activity against non-cancer CCD841 cells. In addition, this hybrid suppressed CRC growth via inhibition of the cell cycle in the G1 phase. This compound **4** also induced apoptosis by activating caspase 3 and PARP cleavage as well as increasing ROS in CRC cells. Finally, hybrid **4** significantly caused a delay in cell growth in CRC using a mouse xenograft model (31). On the other hand, hybrid **5** (hybrids of β-carboline and salicylic acid ) inhibited the proliferation of liver cancer SMMC-7721 cells but not in normal liver LO2 cells, displaying selective inhibition and inducing cancer cell apoptosis (32) (Figure 1). 

**Figure 2:**
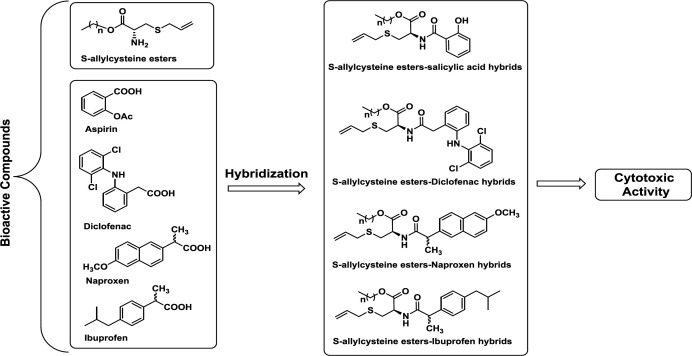
Design of hybrids of *S*-allyl Cysteine methyl ester-based non-steroidal anti-inflammatory drugs as anti-cancer agents

We have synthesized several *S*-allyl cysteine alkyl esters-anti-inflammatory compound hybrids (Figure 2) . Their effect on proliferation was determined to identify possible therapeutic approaches for the treatment of colorectal cancer. 

## Experimental

*Chemical synthesis*

General remarks

^1^H and ^13^C NMR spectra were recorded on a Varian instrument operating at 300 and 75 MHz, respectively. The signals of the deuterated solvent (DMSO-D_6_) were used as reference. Chemical shifts (δ) are expressed in ppm with the solvent peak as reference and TMS as an internal standard; coupling constants (J) are given in Hertz (Hz). Carbon atom types (C, CH, CH_2 _and CH_3_) were determined using the DEPT pulse sequence. High-resolution mass spectra were recorded using electrospray ionization mass spectrometry (ESI-MS). A QTOF Premier instrument with an orthogonal Z-spray-electrospray interface (Waters, Manchester, UK) was used operating in the W-mode. Silica gel 60 (0.063–0.200 mesh, Merck, Whitehouse Station, NJ, USA) was used for column chromatography, and precoated silica gel plates (Merck 60 F254 0.2 mm) were used for thin-layer chromatography (TLC). Monitoring of the reaction progress and product purification was carried out by TLC. 

*Procedure for the synthesis of S-Allyl cysteine (* ***7*** *)*

 S-Cysteine hydrochloride (1 g, 6.34 mmol) was added to allyl bromide (1.15 g, 823 µL, 9.51 mmol) in 2M NH_4_OH (20 mL). The resulting mixture was stirred at room temperature for 20h. Then, the reaction mixture was concentrated to precipitate the product as a white solid. The solid was filtered, washed with ethanol (3-10 mL) and dried under reduced pressure, affording 818 mg (80%) of compound **2**. This compound was used in the following step without further purification.

^1^H NMR (DMSO-D_6_, 300 MHz): δ 2.48 (NH_2_), 2.72 (1H, dd, *J* = 14.6, 8.0 Hz, S-C**H**_2_CHN), 2.87 (1H, dd, *J* = 14.6, 4.2 Hz, S-C**H**_2_CHN), 3.06 (2H, d, *J* = 7.3 Hz, S-C**H**_2_CH=CH_2_), 3.56 (1H, dd, *J* = 8.0, 4.2 Hz, -C**H**-N), 4.29 (-NH_2_), 4.98-5.13 (2H, m, S-CH_2_CH=C**H**_2_), 5.59-5.75 (2H, m, S-CH_2_C**H**=CH_2_); ^13^C NMR (CDCl_3_, 75 MHz): δ 31.33 (S-**C**H_2_CHN), 33.93 (S-**C**H_2_CH=CH_2_), 53.45 (**C**H-N), 118.48 (S-CH_2_CH=**C**H_2_), 133.77 (S-CH_2_**C**H=CH_2_), 171.33 (-C=O).

*Procedure for the synthesis of S-Allyl cysteine methyl ester (* ***8a*** *)*

Thionyl chloride (442.6 mg, 3.72 mmol, 270 µL) was added over 5 min. to dry methanol (15 mL) cooled to -10 °C and the resulting solution stored for a further 5 min. Then *S-*allyl cysteine (500 mg, 3.1 mmol) was added, and the mixture was stirred for 10 min. The resulting solution was stored at -10 °C for 2 h, kept at room temperature for a further 24 h and then poured into ether (100 mL) and refrigerated for 2 h. The product (488 mg, 90%) separated as colorless needles and was removed by filtration.

m.p. 114-116 °C; [α]^25^ + 4.778 (C = 0,013, CHCl_3_); ^1^H NMR (DMSO-D_6_, 300 MHz): δ 2.51 (NH_2_), 2.84 (1H, dd, *J* = 14.7, 5.0 Hz, S-C**H**_2_CHN), 2.93 (1H, dd, *J* = 14.7, 5.0 Hz, S-C**H**_2_CHN), 3.09 (2H, d, *J* = 7.3 Hz, S-C**H**_2_CH=CH_2_), 3.70 (3H, s, OCH_3_), 4.14 (1H, dd, *J* = 7.1, 5.0 Hz, -C**H**-N), 5.04-5.15 (2H, m, S-CH_2_CH=C**H**_2_), 5.59-5.77 (1H, m, S-CH_2_C**H**=CH_2_); ^13^C NMR (CDCl_3_, 75 MHz): δ 30.09 (S-**C**H_2_CHN), 34.20 (S-**C**H_2_CH=CH_2_), 52.09 (O**C**H_3_), 54.52 (**C**H-N), 118.86 (S-CH_2_CH=**C**H_2_), 133.37 (S-CH_2_**C**H=CH_2_), 168.96 (-C=O).

*General procedure for the synthesis of S-Allyl cysteine esters (* ***8b and 8c*** *)*

Thionyl chloride (3 eq) was added over 5 min. to dry ethyl or propyl alcohol (15 mL) cooled to -10 °C, and the resulting solution was stored for a further 5 min. Then, *S*-allyl cysteine (500 mg, 3.1 mmol) was added, and the resulting mixture was stored at -10 °C for 2 h and kept at room temperature for a further 24 h. Then, the excess alcohol was removed by distillation. The residue was purified by column chromatography over silica gel eluting with dichloromethane-methanol (95:5 ratio) to obtain *S*-allyl cysteine ethyl ester and *S*-allyl cysteine propyl ester in 60% (352 mg) and 71% (447 mg) yields, respectively. Monitoring of the reaction progress and product purification was carried out by TLC.

*Ethyl S-prop-2-en-1-ylcysteinate (* ***3b*** *)*

m.p. 121-123 °C; [α]^25^ + 1.40 (C = 0,62, CHCl_3_); ^1^H NMR (CDCl_3_, 600 MHz): δ 0.96 (3H, t, *J* = 7.2 Hz), 2.63 (NH_2_), 3.22-3.25 (2H, m, S-C**H**_2_CHN), 3.18-3.22 (2H, m, S-C**H**_2_CH=CH_2_), 3.29 (1H, dd, *J* = 7.5, 5.0 Hz, -C**H**-N), 4.29 (2H, q, *J* = 7.0 Hz, OCH_2_), 5.15 (1H, d, *J* = 10 Hz, S-CH_2_CH=C**H**_2_), 5.24 (1H, d, *J* = 18 Hz, S-CH_2_CH=C**H**_2_), 5.74-5.85 (1H, m, S-CH_2_C**H**=CH_2_); ^13^C NMR (CDCl_3_, 125 MHz): δ 14.05 (CH_3_), 30.48 (S-**C**H_2_CHN), 35.10 (S-**C**H_2_CH=CH_2_), 52.69 (**C**H-N), 62.94 (OCH_2_), 118.51 (S-CH_2_CH=**C**H_2_), 133.37 (S-CH_2_**C**H=CH_2_), 167.95 (-C=O). 

*Propyl S-prop-2-en-1-ylcysteinate (* ***3c*** *)*

m.p. 115-117 °C; [α]^25^ + 3.750 (C = 0,015, CHCl_3_); ^1^H NMR (CDCl_3_, 300 MHz): δ 0.91 (3H, t, *J* = 7.5 Hz), 1.53-1.72 (2H, m), 1.98 (NH_2_), 2.83 (1H, dd, *J* = 13.5, 5.0 Hz, S-C**H**_2_CHN), 2.65 (1H, dd, *J* = 13.5, 5.0 Hz, S-C**H**_2_CHN), 3.11 (2H, d, *J* = 7.0 Hz, S-C**H**_2_CH=CH_2_), 3.58 (1H, dd, *J* = 7.4, 5.0 Hz, -C**H**-N), 4.05 (2H, t, *J* = 6.7 Hz, OCH_2_), 5.07-5.16 (2H, m, S-CH_2_CH=C**H**_2_), 5.64-5.82 (1H, m, S-CH_2_C**H**=CH_2_); ^13^C NMR (CDCl_3_, 75 MHz): δ 10.41 (CH_3_), 21.97 (CH_2_), 35.14 (S-**C**H_2_CHN), 35.82 (S-**C**H_2_CH=CH_2_), 54.11 (**C**H-N), 66.82 (OCH_2_), 117.61 (S-CH_2_CH=**C**H_2_), 134.01 (S-CH_2_**C**H=CH_2_), 174.15 (-C=O).

*General procedure for condensation using HBTU*

A solution of carboxylic acid (salicylic acid, diclofenac, naproxen and ibuprofen) (1 mmol) and triethylamine (4 mmol) in THF (10 mL) was stirred for 15 min. Then, HBTU (1.5 mmol) was added, and the resulting mixture was stirred for 10 min. Then, *S*-allyl cysteine ester (**8a-8c**) (1.2 mmol) was added, and the resulting mixture was allowed to stir for 15 h. The solvent was removed under reduced pressure, and the residue was purified by chromatography on silica gel afforded compounds **9-12** in yields ranging 25-75%. 

*Methyl S-allyl-N-(2-hydroxybenzoyl)-L-cysteinate (* ***9a*** *)*

Yield 35%, yellow solid; m.p. 112-114 °C; ^1^H NMR (300 MHz, DMSO-*d*_6_) δ 9.82 (s, OH), 7.87 (dd, *J* = 7.9, 1.8 Hz, Ar-H), 7.32 (ddd, *J* = 8.6, 7.1, 1.8 Hz, Ar-H), 6.95 (dd, *J* = 8.3, 1.1 Hz, Ar-H), 6.79 (t, *J* = 7.3 Hz, Ar-H), 5.74 (ddt, *J* = 17.1, 9.9, 7.2 Hz, S-CH_2_C**H**=CH_2_), 5.17 – 5.05 (m, -C**H**-N, S-CH_2_CH=C**H**_2_), 4.76 (t, *J* = 6.4 Hz, -CH-N**H**-C=O), 3.68 (s, OCH_3_), 3.19 (dd, *J* = 7.1, 2.7 Hz, S-C**H**_2_CH=CH_2_), 2.99 (dd, *J* = 13.8, 5.2 Hz, S-C**H**_2_CHN), 2.90 (dd, *J* = 13.8, 7.5 Hz, S-C**H**_2_CHN). ^13^C NMR (75 MHz, DMSO-*d*_6_) δ 171.60 (CH-**C**=O)-O), 167.72 (-NH-**C**=O), 161.21 (Ar), 134.58 (2C-Ar), 133.84 (S-CH_2_**C**H=CH_2_), 129.77 (Ar), 118.34 (S-CH_2_CH=**C**H_2_), 118.06 (Ar), 116.96 (Ar), 52.71 (OCH_3_), 52.51(**C**H-N), 34.49 (S-**C**H_2_CH=CH_2_), 31.78 (S-**C**H_2_CHN). ESI–MS: m/z 296.0956 [M + H]^+^, Calc. For C_14_H_18_NO_4_S: 296.0951.

*Ethyl S-allyl-N-(2-hydroxybenzoyl)-L-cysteinate (* ***9b*** *)*

Yield 30%, yellow oil; ^1^H NMR (300 MHz, DMSO-*d*_6_) δ 7.91 (dd, *J* = 7.9, 1.8 Hz, Ar-H), 7.36 (ddd, *J* = 8.6, 7.1, 1.7 Hz, Ar-H), 7.02 – 6.94 (m, Ar-H), 6.85 (t, *J* = 7.5 Hz, Ar-H), 5.82 – 5.65 (m, S-CH_2_C**H**=CH_2_), 5.17 – 5.04 (m, (m, -C**H**-N, S-CH_2_CH=C**H**_2_), 4.69 (d, *J* = 7.2 Hz, -CH-N**H**-C=O), 4.14 (q, *J* = 6.9 Hz, -OC**H**_2_CH_3_), 3.18 (dd, *J* = 7.1, 1.2 Hz, S-C**H**_2_CH=CH_2_), 2.99 (dd, *J* = 13.9, 5.2 Hz, S-C**H**_2_CHN), 2.91 (dd, *J* = 13.9, 7.7 Hz, S-C**H**_2_CHN), 1.20 (t, *J* = 7.1 Hz, CH_2_C**H**_3_). ^13^C NMR (75 MHz, DMSO-d6) δ 170.95 (CH-**C**=O)-O), 167.78 (-NH-**C**=O), 162.63 (Ar), 134.56 (2C-Ar), 134.01 (S-CH_2_**C**H=CH_2_), 129.71 (Ar), 118.57 (S-CH_2_CH=**C**H_2_), 118.06 (Ar), 116.66 (Ar), 115.84 (Ar), 61.46 (OCH_2_CH_3_), 52.63 (**C**H-N), 34.44 (S-**C**H_2_CH=CH_2_), 31.63 (S-**C**H_2_CHN), 14.51 (CH_3_). ESI–MS: m/z 310.1117 [M + H]^+^, Calc. For C_15_H_20_NO_4_S: 310.1107.

*Propyl S-allyl-N-(2-hydroxybenzoyl)-L-cysteinate (* ***9c*** *)*

Yield 75%, yellow oil; ^1^H NMR (300 MHz, DMSO-d6) δ 9.14 (s, OH), 7.77 (dd, *J* = 8.0, 1.6 Hz, Ar-H), 7.25 (ddd, *J* = 8.5, 7.2, 1.7 Hz, Ar-H), 6.83 (d, *J* = 7.5 Hz, Ar-H), 6.75 (t, *J* = 7.5 Hz, Ar-H), 5.62 (tdt, *J* = 17.1, 9.9, 7.4 Hz, S-CH_2_C**H**=CH_2_), 5.02 – 4.90 (m, -C**H**-N, S-CH_2_CH=C**H**_2_), 4.55 (q, *J* = 7.3 Hz, -CH-N**H**-C=O), 3.90 (q, *J* = 6.5 Hz, -OC**H**_2_CH_3_), 3.03 (d, *J* = 7.4 Hz, S-C**H**_2_CH=CH_2_), 2.85 (dd, *J* = 13.9, 5.2 Hz, S-C**H**_2_CHN), 2.77 (dd, *J* = 13.8, 7.9 Hz, S-C**H**_2_CHN), 1.44 (q, *J* = 6.9 Hz, C**H**_2_CH_3_), 0.72 (t, *J* = 7.4 Hz, CH_2_C**H**_3_). ^13^C NMR (75 MHz, DMSO-*d*_6_) δ 170.92 (CH-**C**=O)-O), 167.94 (-NH-**C**=O), 162.72 (Ar), 134.57 (2C-Ar), 134.22 (S-CH_2_**C**H=CH_2_), 129.56 (Ar), 118.05 (S-CH_2_CH=**C**H_2_), 118.0 (Ar), 117.77 (Ar), 116.40 (Ar), 66.86 (OCH_2_CH_3_), 52.68 (**C**H-N), 34.41 (S-**C**H_2_CH=CH_2_), 31.52 (S-**C**H_2_CHN), 21.94 (CH_2_), 10.68 (CH_3_). ESI–MS: m/z 324.1264 [M + H]^+^, Calc. For C_16_H_22_NO_4_S: 324.1264.

*Methyl S-allyl-N-(2-(2-((2,6-dichlorophenyl)amino)phenyl)acetyl)-L-cysteinate (* ***10a*** *)*

Yield 30%, red solid; m.p. 90-91 °C; ^1^H NMR (300 MHz, DMSO-*d*_6_) δ 8.95 (d, *J* = 7.8 Hz, -CH-N**H**-C=O), 8.00 (s, Ar-**NH**-Ar), 7.52 (d, *J* = 8.1 Hz, 2H-Ar), 7.23 (dd, *J* = 7.6, 1.6 Hz, Ar), 7.17 (t, *J* = 8.1 Hz, Ar), 7.04 (td, *J* = 7.7, 1.6 Hz, Ar), 6.89 – 6.81 (m, Ar), 6.28 (d, *J* = 8.0 Hz, Ar), 5.79 – 5.61 (m, S-CH_2_C**H**=CH_2_), 5.11 – 5.00 (m, S-CH_2_CH=C**H**_2_), 4.59 – 4.46 (m, -C**H**-N), 3.67 (s, Ar-C**H**_2_-C=O), 3.64 (s, OCH_3_), 3.13 (d, *J* = 7.1 Hz, S-C**H**_2_CH=CH_2_), 2.83 (dd, *J* = 13.8, 5.5 Hz, S-C**H**_2_CHN), 2.73 (dd, *J* = 13.9, 7.8 Hz, S-C**H**_2_CHN). ^13^C NMR (75 MHz, DMSO-*d*_6_) δ 172.05 (-NH-**C**=O), 171.40 (CH-**C**=O)-O), 143.37 (Ar), 137.59 (Ar), 134.55 (S-CH_2_**C**H=CH_2_), 130.96 (Ar), 130.10 (2C-Ar), 129.66 (2C-Ar), 127.77 (Ar), 125.72 (Ar), 121.14 (Ar), 118.02 (S-CH_2_CH=**C**H_2_), 116.34 (Ar), 52.65 (OCH_3_), 52.52 (**C**H-N), 37.98 (Ar-**C**H_2_-C=O), 34.56 (S-**C**H_2_CH=CH_2_), 31.73 (S-**C**H_2_CHN). ESI–MS: m/z 453.0831 [M + H]^+^, Calc. For C_21_H_23_Cl_2_N_2_O_3_S: 453.0801.

*Ethyl S-allyl-N-(2-(2-((2,6-dichlorophenyl)amino)phenyl)acetyl)-L-cysteinate (* ***10b*** *)*

Yield 32%, yellow solid; m.p. 88-90 °C; ^1^H NMR (300 MHz, DMSO-*d*_6_) δ 8.94 (d, *J* = 7.7 Hz, -CH-N**H**-C=O), 8.02 (s, Ar-**NH**-Ar), 7.52 (d, *J* = 8.1 Hz, 2H-Ar), 7.29 – 7.20 (m, Ar), 7.16 (t, *J* = 8.0 Hz, Ar), 7.04 (t, *J* = 7.2 Hz, Ar), 6.96 (d, *J* = 8.6 Hz, Ar), 6.90 – 6.79 (m, S-CH_2_C**H**=CH_2_), 6.28 (d, *J* = 7.9 Hz, Ar), 5.12 – 5.00 (m, S-CH_2_CH=C**H**_2_), 4.47 (td, *J* = 7.7, 5.4 Hz, -C**H**-N), 4.09 (q, J = 7.13 Hz, OC**H**_2_CH_3_), 3.67 (s, Ar-C**H**_2_-C=O), 3.14 (d, *J* = 7.1 Hz, S-C**H**_2_CH=CH_2_), 2.83 (dd, *J* = 13.8, 5.4 Hz, S-C**H**_2_CHN), 2.73 (dd, *J* = 13.8, 7.9 Hz, S-C**H**_2_CHN), 1.13 (t, *J* = 7.1 Hz, CH_3_). ^13^C NMR (75 MHz, DMSO-*d*_6_) δ 172.08 (-NH-**C**=O), 170.86 (CH-**C**=O)-O), 143.37 (Ar), 137.60 (Ar), 134.53 (S-CH_2_**C**H=CH_2_), 130.95 (Ar), 130.09 (2C-Ar), 129.66 (2C-Ar), 127.75 (Ar), 125.71 (Ar), 121.11 (Ar), 118.02 (S-CH_2_CH=**C**H_2_), 116.33 (Ar), 61.34 (OCH_2_), 55.92 (**C**H-N), 36.67 (Ar-**C**H_2_-C=O), 34.55 (S-**C**H_2_CH=CH_2_), 31.69 (S-**C**H_2_CHN), 14.38 (CH_3_). ESI–MS: m/z 467.0956 [M + H]^+^, Calc. For C_22_H_25_Cl_2_N_2_O_3_S: 467.0957.

*Propyl S-allyl-N-(2-(2-((2,6-dichlorophenyl) amino)phenyl)acetyl)-L-cysteinate (* ***10c*** *)*

Yield 60%, red oil; ^1^H NMR (600 MHz, DMSO-*d*_6_) δ 8.90 (d, *J* = 7.8 Hz, -CH-N**H**-C=O), 8.02 (s, Ar-**NH**-Ar), 7.52 (d, *J* = 8.1 Hz, 2H-Ar), 7.23 (dd, *J* = 7.6, 1.6 Hz, Ar), 7.17 (t, *J* = 8.1 Hz, Ar), 7.06 – 7.02 (m, Ar), 6.85 (td, *J* = 7.4, 1.2 Hz, S-CH_2_C**H**=CH_2_), 6.31 – 6.26 (m, Ar), 5.11 – 5.03 (m, S-CH_2_CH=C**H**_2_), 4.49 (td, *J* = 7.9, 5.4 Hz, -C**H**-N), 4.09 (q, J = 7.13 Hz, OC**H**_2_), 3.67 (s, Ar-C**H**_2_-C=O), 3.17 – 3.13 (m, S-C**H**_2_CH=CH_2_), 2.83 (dd, *J* = 13.8, 5.5 Hz, S-C**H**_2_CHN), 2.74 (dd, *J* = 13.8, 8.0 Hz, S-C**H**_2_CHN), 1.58 – 1.49 (m, CH_2_), 0.84 (t, *J* = 7.4 Hz, CH_3_). ^13^C NMR (151 MHz, DMSO-*d*_6_) δ 172.07 (-NH-**C**=O), 170.93 (CH-**C**=O)-O), 143.40 (Ar), 137.64 (Ar), 134.55 (S-CH_2_**C**H=CH_2_), 130.95 (Ar), 130.06 (2C-Ar), 129.67 (2C-Ar), 127.76 (Ar), 125.49 (Ar), 121.15 (Ar), 118.00 (S-CH_2_CH=**C**H_2_), 116.40 (Ar), 66.74 (OCH_2_), 52.69 (**C**H-N), 34.56 (Ar-**C**H_2_-C=O), 31.72 (S-**C**H_2_CH=CH_2_), 29.99 (S-**C**H_2_CHN), 21.88 (CH_2_), 10.65 (CH_3_). ESI–MS: m/z 481.1120 [M + H]^+^, Calc. For C_23_H_27_Cl_2_N_2_O_3_S: 481.1114

*Methyl S-allyl-N-(2-(6-methoxynaphthalen-2-yl)propanoyl)-L-cysteinate (* ***11a*** *)*

Yield 60%, yellow oil; ^1^H NMR (300 MHz, DMSO-*d*_6_) δ 8.56 (d, *J* = 7.4 Hz, -CH-N**H**-C=O), 7.83 – 7.68 (m, 3H-Ar), 7.45 (t, *J* = 6.8 Hz, Ar), 7.27 (s, Ar), 7.20 – 7.09 (m, Ar), 5.83 – 5.51 (m, S-CH_2_C**H**=CH_2_), 5.16 – 4.89 (m, S-CH_2_CH=C**H**_2_), 4.53 – 4.37 (m, -C**H**-N), 3.86 (s, OCH_3_), 3.64 (3.55) (s, OCH_3_), 3.15 (3.01) (d, *J* = 7.2 Hz, S-C**H**_2_CH=CH_2_), 2.84 (dd, *J* = 13.3, 5.7 Hz, S-C**H**_2_CHN), 2.71 (dd, *J* = 15.2, 7.6 Hz, S-C**H**_2_CHN), 1.41 (d, *J* = 6.9 Hz, CHC**H**_3_). ^13^C NMR (75 MHz, DMSO-*d*_6_) δ 174.08 (174.04) (-NH-**C**=O), 171.70 (171.59) (CH-**C**=O)-O), 157.45 (Ar), 137.36 (Ar), 134.64 (134.50) (Ar), 133.60 (S-CH_2_**C**H=CH_2_), 129.56 (Ar), 128.80 (Ar), 127.07 (126.99) (2C-Ar), 125.83 (Ar), 119.02 (Ar), 117.95 (117.81) (S-CH_2_CH=**C**H_2_), 106.10 (Ar), 55.60 (OCH_3_), 52.45 (52.55) (**C**H-N), 52.29 (OCH_3_), 44.95 (**C**H-CH_3_), 34.44 (S-**C**H_2_CH=CH_2_), 31.74 (31.65) (S-**C**H_2_CHN), 19.14 (18.94) (CH-**C**H_3_). ESI–MS: m/z 388.1580 [M + H]^+^, Calc. For C_21_H_26_NO_4_S: 388.1577

*Ethyl S-allyl-N-(2-(6-methoxynaphthalen-2-yl)propanoyl)-L-cysteinate (* ***11b*** *)*

Yield 25%, yellow oil; ^1^H NMR (300 MHz, DMSO-*d*_6_) δ 8.55 (dd, *J* = 7.9, 3.1 Hz, -CH-N**H**-C=O), 7.82 – 7.69 (m, 3H-Ar), 7.45 (ddd, *J* = 8.5, 4.4, 1.7 Hz, Ar), 7.28 (s_app_, Ar), 7.14 (ddd, *J* = 8.9, 2.6, 1.1 Hz, Ar), 5.82 – 5.50 (m, S-CH_2_C**H**=CH_2_), 5.18 – 4.89 (m, S-CH_2_CH=C**H**_2_), 4.15 – 4.04 (m, -C**H**-N), 3.99 (q, *J* = 7.20 Hz, OCH_2_CH_3_), 3.85 (s, OC**H**_2_CH_3_), 3.15 (3.02) (d, *J* = 7.1 Hz, S-C**H**_2_CH=CH_2_), 2.83 (dd, *J* = 13.8, 5.4 Hz, S-C**H**_2_CHN), 2.66 (dd, *J* = 13.7, 8.2 Hz, S-C**H**_2_CHN), 1.41 (d, *J* = 7.0 Hz, CHC**H**_3_), 1.16 (1.04) (t, *J* = 7.1 Hz, OCH_2_C**H**_3_). ^13^C NMR (75 MHz, DMSO-*d*_6_) δ 174.10 (173.99) (-NH-**C**=O), 171.14 (171.04) (CH-**C**=O)-O), 157.44 (Ar), 137.36 (137.34) (Ar), 134.62 (134.49) (Ar), 133.61 (S-CH_2_**C**H=CH_2_), 129.55 (Ar), 128.80 (Ar), 127.02 (126.97) (2C-Ar), 125.84 (125.82) (Ar), 119.01(Ar), 117.94 (117.82) (S-CH_2_CH=**C**H_2_), 106.10 (Ar), 61.22 (61.11) (CH_3_**C**H_2_O), 55.59 (OCH_3_), 52.58 (52.36) (**C**H-N), 44.94 (**C**H-CH_3_), 34.44 (S-**C**H_2_CH=CH_2_), 31.70 (31.61) (S-**C**H_2_CHN), 19.03 (18.94) (CH-**C**H_3_), 14.44 (14.32) (OCH_2_**C**H_3_). ESI–MS: m/z 402.1737 [M + H]^+^, Calc. For C_22_H_28_NO_4_S: 402.1733.

*Propyl S-allyl-N-(2-(6-methoxynaphthalen-2-yl)propanoyl)-L-cysteinate (* ***11c*** *)*

Yield 62%, yellow oil; ^1^H NMR (300 MHz, DMSO-*d*_6_) δ 8.55 (dd, *J* = 7.9, 3.9 Hz, -CH-N**H**-C=O), 7.81 – 7.69 (m, 3H-Ar), 7.45 (ddd, *J* = 8.4, 5.1, 1.7 Hz, Ar), 7.27 (d, *J* = 2.5 Hz, Ar), 7.13 (dd, *J* = 9.0, 2.6 Hz, Ar), 5.82 – 5.53 (m, S-CH_2_C**H**=CH_2_), 5.17 – 4.89 (m, S-CH_2_CH=C**H**_2_), 4.50 – 4.35 (m, -C**H**-N), 4.08 – 3.93 (m, OC**H**_2_CH_3_), 3.85 (s, OCH_3_), 3.16 (3.03) (d, *J* = 7.2 Hz, S-C**H**_2_CH=CH_2_), 2.84 (dd, *J* = 13.8, 5.5 Hz, S-C**H**_2_CHN), 2.72 (dd, *J* = 12.1, 5.2 Hz, S-C**H**_2_CHN), 1.63-1.48 (m, CH_3_C**H**_2_CH_2_O), 1.41 (d, *J* = 6.0 Hz, CHC**H**_3_), 0.85 (0.72) (t, *J* = 7.4 Hz, OCH_2_C**H**_3_). ^13^C NMR (75 MHz, DMSO-*d*_6_) δ 174.08 (173.95) (-NH-**C**=O), 171.21 (171.10) (CH-**C**=O)-O), 157.42 (Ar), 137.32 (Ar), 134.62 (134.48) (Ar), 133.61(S-CH_2_**C**H=CH_2_), 129.53 (Ar), 128.79 (Ar), 126.96 (125.81) (2C-Ar), 118.99 (Ar), 117.79 (117.90) (S-CH_2_CH=**C**H_2_), 106.08 (Ar), 66.61 (66.51) (CH_3_**C**H_2_O), 55.58 (OCH_3_), 52.55 (52.32) (**C**H-N), 44.95 (**C**H-CH_3_), 34.41(S-**C**H_2_CH=CH_2_), 31.80 (31.59) (S-**C**H_2_CHN), 21.91 (CH_3_**C**H_2_CH_2_O), 19.02 (18.91) (CH-**C**H_3_), 10.67 (10.54) (**C**H_3_CH_2_CH_2_O). ESI–MS: m/z 416.1891 [M + H]^+^, Calc. For C_23_H_30_NO_4_S: 416.1890.

*Methyl S-allyl-N-(2-(4-isobutylphenyl)propanoyl)-L-cysteinate (* ***12a*** *)*

Yield 31%, yellow solid; m.p. 89-91 °C; ^1^H NMR (300 MHz, DMSO-*d*_6_) δ 8.49 (dd, *J* = 7.8, 2.6 Hz, -CH-N**H**-C=O), 7.22 (dd, *J* = 8.0, 5.8 Hz, 2H-Ar), 7.07 (dd, *J* = 8.2, 2.8 Hz, 2H-Ar), 5.82 – 5.53 (m, S-CH_2_C**H**=CH_2_), 5.17 – 4.91 (m, S-CH_2_CH=C**H**_2_), 4.49 – 4.37 (m, -C**H**-N), 3.74 – 3.65 (m, C**H**-C=O), 3.63 (3.57) (s, OCH_3_), 3.14 (3.01) (d, *J* = 7.2 Hz, S-C**H**_2_CH=CH_2_), 2.83 (dd, *J* = 13.8, 5.5 Hz, S-C**H**_2_CHN), 2.74 (dd, *J* = 14.0, 5.5 Hz, S-C**H**_2_CHN), 2.39 (d, *J* = 7.2 Hz, Ar-C**H**_2_-CH(CH_3_)_2_), 1.86 – 1.70 (m, CH_2_-C**H**(CH_3_)_2_), 1.30 (dd, *J* = 7.0, 2.4 Hz, CH-C**H**_3_), 0.85 (d, *J* = 6.6 Hz, CH(C**H**_3_)_2_), 0.84 (d, *J* = 6.6 Hz, CH(C**H**_3_)_2_). ^13^C NMR (75 MHz, DMSO-d6) δ 174.12 (174.07) (-NH-**C**=O), 171.68 (171.57) (CH-**C**=O)-O), 139.67 (139.70) (Ar), 139.48 (139.45) (Ar), 134.63 (134.51) (S-CH_2_**C**H=CH_2_), 129.16 (2C-Ar), 127.52 (2C-Ar), 117.93 (117.83) (S-CH_2_CH=**C**H_2_), 52.51 (**C**H-N), 52.41 (52.26) (OCH_3_), 44.70 (**C**H-CH_3_), 44.61 (Ar-C**H**_2_-CH(CH_3_)_2_), 34.49 (34.44) (S-**C**H_2_CH=CH_2_), 31.74 (31.58) (S-**C**H_2_CHN), 30.11(Ar-**C**H-(CH_3_)_2_), 22.63 (CH-(**C**H_3_)_2_), 19.19 (18.98) (CH-**C**H_3_). ESI–MS: m/z 364.1952 [M + H]^+^, Calc. For C_20_H_30_NO_3_S: 364.1940.

*Ethyl S-allyl-N-(2-(4-isobutylphenyl)propanoyl)-L-cysteinate (* ***12b*** *)*

Yield 42%, yellow solid; m.p. 65-67 °C; ^1^H NMR (300 MHz, DMSO-*d*_6_) δ 8.48 (d, *J* = 7.8 Hz, -CH-N**H**-C=O), 7.22 (dd, *J* = 7.9, 5.2 Hz, 2H-Ar), 7.07 (d, *J* = 7.8 Hz, 2H-Ar), 5.84 – 5.55 (m, S-CH_2_C**H**=CH_2_), 5.19 – 4.91 (m, S-CH_2_CH=C**H**_2_), 4.47 – 4.30 (m, -C**H**-N), 4.01 (q, *J* = 7.2 Hz, OC**H**_2_CH_3_), 3.75 – 3.62 (m, C**H**-C=O), 3.14 (3.03) (d, *J* = 7.2 Hz, S-C**H**_2_CH=CH_2_), 2.82 (dd, *J* = 13.9, 5.6 Hz, S-C**H**_2_CHN), 2.70 (dd, *J* = 13.8, 5.9 Hz, S-C**H**_2_CHN), 2.39 (d, *J* = 7.1 Hz, Ar-C**H**_2_-CH(CH_3_)_2_), 1.89 – 1.71 (m, CH_2_-C**H**(CH_3_)_2_), 1.30 (dd, *J* = 7.1, 1.3 Hz, CH-C**H**_3_), 1.16 (1.08) (t, *J* = 7.1 Hz, OCH_2_C**H**_3_), 0.84 (dd, *J* = 6.6, 1.1 Hz, CH(C**H**_3_)_2_). ^13^C NMR (75 MHz, DMSO-*d*_6_) δ 174.13 (174.0) (-NH-**C**=O), 171.13 (171.03) (CH-**C**=O)-O), 139.71 (139.67) (Ar), 139.47 (139.45) (Ar), 134.62 (134.52) (S-CH_2_**C**H=CH_2_), 129.16 (2C-Ar), 127.53 (127.50) (2C-Ar), 117.93 (117.84) (S-CH_2_CH=**C**H_2_), 61.19 (61.10) (O**C**H_2_CH_3_), 52.62 (52.34) (**C**H-N), 44.69 (Ar-C**H**_2_-CH(CH_3_)_2_), 44.62 (**C**H-CH_3_), 34.49 (34.44) (S-**C**H_2_CH=CH_2_), 31.54 (31.68) (S-**C**H_2_CHN), 30.12 (**C**H-(CH_3_)_2_), 22.62 (CH-(**C**H_3_)_2_), 19.08 (18.97) (CH-**C**H_3_), 14.44 (14.34) (CH_2_C**H**_3_). ESI–MS: m/z 378.2099 [M + H]^+^, Calc. For C_21_H_32_NO_3_S: 378.2097

*Propyl S-allyl-N-(2-(4-isobutylphenyl)propanoyl)-L-cysteinate (* ***12c*** *)*

Yield 40%, yellow solid; m.p. 58-60 °C; ^1^H NMR (300 MHz, DMSO-*d*_6_) δ 8.47 (d, *J* = 7.7 Hz, -CH-N**H**-C=O), 7.22 (dd, *J* = 7.8, 5.7 Hz, 2H-Ar), 7.06 (d, *J* = 7.8 Hz, 2H-Ar), 5.79 – 5.54 (m, S-CH_2_C**H**=CH_2_), 5.17 – 4.88 (m, S-CH_2_CH=C**H**_2_), 4.39 (ddd, *J* = 13.1, 8.5, 5.4 Hz, -C**H**-N), 4.00 (q, *J* = 6.3 Hz, OC**H**_2_CH_3_), 3.74 – 3.62 (m, C**H**-C=O), 3.14 (3.03) (d, *J* = 7.1 Hz, S-C**H**_2_CH=CH_2_), 2.82 (dd, *J* = 13.9, 5.7 Hz, S-C**H**_2_CHN), 2.71 (dd, *J* = 13.2, 6.5 Hz, S-C**H**_2_CHN), 2.39 (d, *J* = 7.1 Hz, Ar-C**H**_2_-CH(CH_3_)_2_), 1.87-1.71 (m, CH_2_-C**H**(CH_3_)_2_),1.62-1.40 (m, CH_3_C**H**_2_CH_2_O),1.30 (d, *J* = 7.0 Hz, CH-C**H**_3_), 0.89 – 0.74 (m, OCH_2_C**H3**, CH(C**H**_3_)_2_). ^13^C NMR (75 MHz, DMSO-*d*_6_) δ 174.13 (173.97) (-NH-**C**=O), 171.21 (171.11) (CH-**C**=O)-O), 139.70 (139.66) (Ar), 139.47 (139.45) (Ar), 134.62 (134.51) (S-CH_2_**C**H=CH_2_), 129.15 (2C-Ar), 127.53 (127.49) (2C-Ar), 117.90 (117.82) (S-CH_2_CH=**C**H_2_), 66.60 (66.53) (O**C**H_2_CH_3_), 52.61 (52.32) (**C**H-N), 44.70 (Ar-C**H**_2_-CH(CH_3_)_2_), 44.64 (**C**H-CH_3_), 34.47 (34.43) (S-**C**H_2_CH=CH_2_), 31.70 (31.55) (S-**C**H_2_CHN), 30.12 (**C**H-(CH_3_)_2_), 22.62, (CH-(**C**H_3_)_2_), 21.92 (21.85) (OCH_2_**C**H_2_CH_3_), 19.11 (18.98) (CH-**C**H_3_), 10.68 (10.63) (CH_2_C**H**_3_). ESI–MS: m/z 392.2259 [M + H]^+^, Calc. For C_22_H_34_NO_3_S: 392.2254.

*In-vitro biological assays*

*Cell lines and culture medium*

Biological assays were performed using adenocarcinoma colon cancer (SW480) and non-malignant (CHO-K1) cell lines obtained from The European Collection of Authenticated Cell Cultures (ECACC, England). Cells were cultured in 25-cm^2^ flasks containing Dulbecco’s Modified Eagle Medium, supplemented with 10% heat-inactivated (56 °C) horse serum, 1% penicillin/streptomycin and 1% non-essential amino acids (Gibco Invitrogen, Carlsbad, USA). For all experiments, horse serum was reduced to 3%, and the medium was supplemented with 5 mg/mL transferrin, 5 mg/mL selenium and 10 mg/mL insulin (ITS-defined medium; Gibco, Invitrogen, Carlsbad, USA) (33).

*Growth inhibition (SRB)*

The growth inhibition of the ethanol extract, the Chromone and 5-fluorouracil (5-FU; the standard drug) was evaluated through sulforhodamine B (SRB) assay, a colorimetric test that is based on staining of total cellular protein of adherent cells. Cells were seeded to a ﬁnal density of 20.000 cells/well in 96-well tissue culture plates and incubated at 37 °C in a humidified atmosphere at 5% CO_2_. All cultures were allowed to grow for 24 h. Afterward, they were treated with 1% of DMSO (vehicle control) or increasing concentrations (10^-5^–0,2 mM) of the compounds. After treatment, cells were fixed with trichloroacetic acid (50% v/v; MERCK) for one hour at 4 °C. Cell proteins were determined by staining with SRB (0.4% w/v; Sigma-Aldrich, United States), then they were washed with 1% acetic acid to remove unbound SRB and left for air-drying. Protein-bound SRB was solubilized in 10 mM Tris-base, and the absorbance was measured at 492 nm in a microplate reader (Mindray MR-96A) (33, 34). All experiments were performed in quintuplicate. These values were used to calculate the IC_50_, through dose-response curves for each compound and the selectivity index (SI), by the ratio of IC_50_ values in non-malignant CHO-K1 cells to IC_50_ of SW480 cells. 

*Statistical analysis*

All experiments were performed at least three times. Data are reported as mean ± SE (standard error). Statistical differences between the control group (non-treated) and treated cells were evaluated by one-way ANOVA followed by the Dunnett’s test. Values with *p* ≤ 0.05 were considered significant. Data were analyzed with GraphPad Prism version 7.04 for Windows (Graph Pad Software, San Diego, California, USA).

*Computational Methods*

*In-silico Pharmacokinetic and ADME-tox studies*

*In-silico* drug-likeness prediction along with further ADMET (absorption, distribution, metabolism, excretion and toxicity) tools present an array of opportunities that help accelerate the discovery of new anti-cancer candidates. To find out the drug-like properties, the title hybrids **9**-**12** were screened for their pharmacokinetic properties using opensource cheminformatics toolkits such us Molinspiration software (35) (for MW, rotatable bonds and topographical polar surface area (PSA) descriptors, ALOGPS 2.1 algorithm from the Virtual Computational Chemistry Laboratory (for: LogP_o/w_ descriptor), Pre-ADMET 2.0 program to predicted various pharmacokinetic parameters and pharmaceutical relevant properties such as apparent predicted intestinal permeability (App. Caco-2), binding to human serum albumin (LogK_HSA_), MDCK cell permeation coefficients and intestinal or oral absorption (%HIA). These important parameters define absorption, permeability, motion and action of the drug molecule. The interpretation of two predicted ADMET properties using the Pre-ADMET program was as shown below: Value of Caco-2 permeability is classified into three classes: (1) If permeability < 4, low permeability; (2) if permeability < 70, moderate permeability; and (3) if permeability > 70, higher permeability. Value of MDCK cell permeability can be classified into three classes: 1) If permeability < 25, low permeability; 2) if 25 < permeability < 500, moderate permeability; and 3) if permeability > 500, higher permeability. Likewise, the Molinspiration web server was also used to predict the potential interaction of novel compounds with the most common human receptors G protein-coupled receptor (GPCR), ion channel, kinase, nuclear receptor, protease, and enzymes. Next to it, the OSIRIS Property Explorer (free open source) was used to evaluate the overall drug-score and the most common toxicity human parameters like mutagenic, tumorigenic, irritant effect, and possible injuries can affect the reproductive system.

## Results and Discussion

*Chemistry*

**Scheme 1:**
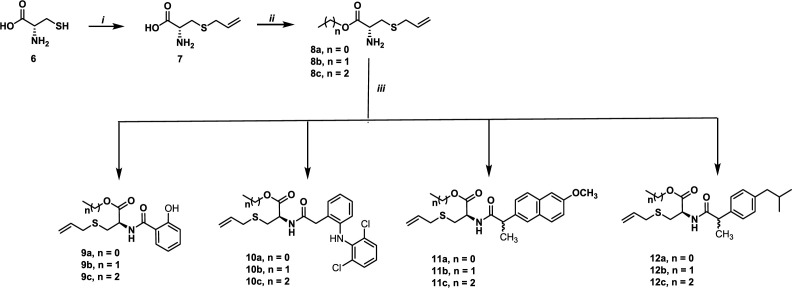
Synthesis of hybrids of *S*-allyl Cysteine methyl ester-based non-steroidal anti-inflammatory drugs. Reagents and conditions: (i) Allyl bromide, NH_4_OH, 80% (ii) SOCl_2_, ROH, -10 °C, 60-90%. (iii) HBTU, Et_3_N, THF, (salicylic acid, diclofenac, naproxen or ibuprofen) between 25-75% yields

Allyl cysteine **7** was obtained, in 80% yield, via nucleophilic substitution between cysteine **6** and allyl bromide (36). The reaction of **7** with the corresponding alcohol in the presence of thionyl chloride (37) afforded, after purification by crystallization or column chromatography, compounds **8a-8c** in 60-90% yields. When these compounds were submitted to peptide type-coupling (38) with salicylic acid, diclofenac, naproxen and ibuprofen, the compounds **9-12** were obtained in 25-75% yields (Scheme 1). 

The structures of all compounds have been established by a combined study of ESI-MS, ^1^H-NMR, ^13^C-NMR, Carbon atom types (C, CH, CH_2_, CH_3_) were determined by using the DEPT or APT pulse sequence. ESI-MS spectra showed characteristic [M+H]^+^ peaks corresponding to their molecular weights. The assignments of all the signals to individual H or C-atoms have been performed based on typical δ-values and *J*-constants. The ^1^H-NMR spectra of hybrids **9-12** dissolved in DMSO showed signals of S-C**H**_2_CHN (2.90 and 3.00 ppm) which appear as *dd*, S-C**H**_2_CH=CH_2 _(3.12 ppm, as a *d*), OCH_2_ or OCH_3_ (4.2 or 3.8, respectively), -C**H**-N (4.86-5.03 ppm, as a *m*), S-CH_2_CH=C**H**_2_ (5.10-5.22 ppm), S-CH_2_C**H**=CH_2_ (5.70-5.88, m), -CH-N**H**-C=O (6.78 ppm, as a *d*) and Ar-H (6.80-7,62 Ar-H). Additionally, compounds **10 and 12** showed the signal corresponding to the methylene group (Ar-C**H**_2_- 3.67 ppm for **10** and 2.39 ppm for **12**). Finally, both hybrids **11a-11c** and **12a-12c** exhibited a doublet to 1.41 and 1.30 ppm, respectively, due to the -CHC**H**_3_ group. ^13^C-NMR spectra of the hybrids showed the signals corresponding to *S*-allyl cysteine esters and aromatic ring: (S-**C**H_2_CHN), (S-**C**H_2_CH=CH_2_), (**C**H-N), (OCH_2_ or OCH_3_), (S-CH_2_CH=**C**H_2_), (S-CH_2_**C**H=CH_2_), (Ar-**C**=C), (-NH-**C**=O) and ((CH-**C**=O)-O). Also, compounds **10 **and** 12 **exhibited the signal corresponding to the methylene group, and hybrids **11** showed the signal duo the isopropylidene group. It is important to note that in the synthesis, we used racemic mixtures of ibuprofen and naproxen. Therefore, duplicate signals are observed in both the proton and carbon.

*Biological activity*

*Cytotoxic effect of S-allyl Cysteine methyl ester-based non-steroidal anti-inflammatory drugs on SW480 and CHO-K1 cell lines*

To determine the cytotoxic effect of the synthesized hybrids, **9a-c** (based on salicylic acid), **10a-c** (based on diclofenac), **11a-c** (based on naproxen) and **12a-c** (based on ibuprofen), these were evaluated against SW480 human colon carcinoma cells and the non-malignant CHO-K1 cell line, through the sulforhodamine B assay. Cytotoxicity was reported as 50% inhibitory concentration (IC_50_ values). 5-FU was used as the reference drug. All results regarding the cytotoxic effect are summarized in Table 1.

Among the tested compounds, it was observed that hybrids **10b-c** together with **11b** and **12b** displayed the best cytotoxic activity with IC_50_ values ranging from 0.131 ± 0.012 to 0.183 ± 0.005 mM on SW480 cells, after 48 h of treatment. These compounds demonstrate the objective of molecular hybridization, improving the biological activity since compounds **10b-c** and **11b** were significantly more active than parental compounds (diclofenac and naproxen, respectively; *p *≤  0.05), and their selectivity indices were comparable to the reference drug (SI > 1). These results are in accordance with those previously reported by Castrillon and colleagues, which also reported cytotoxic and selective activity of different *S*-allyl cysteine hybrids using the same model (39). Moreover, similar results were reported by Herrera-R *et al.* (2018), which also found better cytotoxic activity and selectivity with some styrylcoumarin hybrids when tested using an *in-vitro* model of colorectal cancer (40). Furthermore, although compound **10a** showed the lowest IC_50_ value on cancer cells (0.120 ± 0.004 mM), this value was also low in non-malignant cells (0.112 ± 0.008 mM), and thus the selectivity indices were lower than 1 both in 24 and 48 h after treatment. On the other hand, compound **9b** decreased activity after 48 hours of treatment, evidenced by increased IC_50_ value, with loss in selectivity. The other hybrids evaluated (**9a**, **9c**, **11a**, **11c**, **12a** and **12c**) displayed activity neither 24 h nor 48 h after treatment.

In the structure-activity relationship (SAR) study, excluding the hybrids based on salicylic acid (**9a-c**), which did not show any activity (IC_50_ = >0.20 mM), we proved the synergistic action of the parent subunits when they were linked to form a single structure in the hybrid, as in the case of compounds **10b-c**, **11b**, **12b** *vs.* diclofenac, naproxen and ibuprofen, respectively. In addition, it was possible to denote that compounds with a two-carbon atom alkylic chain displayed the best activity (**10b**, **11b** and **12b**). 

*Pharmacokinetic Studies and In-silico Adme-Tox Modeling*

*Lipinski’s rule and drug-likeness evaluation*

Calculated drug-likeness parameters play a key role in assessing the quality of novel anti-cancer candidates. Early predictions of the pharmacokinetic behavior of the promising oncology compounds based on their structure could help find safer and more effective leads for future preclinical testing. In this work, we screened ten of the most important pharmacokinetic and ADME indices for hybrids **9**-**12** to examine their potential as drug-like (Table 2). These predicted results would reveal the drug ability of the hybrids, demonstrating their potential as a likely orally active anti-cancer option. 

nterestingly, favorable pharmacokinetics indices were found for the synthesized compounds compared to 95% of approved drugs. According to Lipinski’s rule of five (no more than one violation is acceptable) (41), the tested compounds could be promising drug candidates for oral administration. 

An estimated 100% for human intestinal absorption (HIA%) suggests that all hybrids could be absorbed efficiently throughout the intestinal segments upon oral administration. Additionally, high degrees of lipophilicity (calculated as LogP_o/w_) were found for all the compounds (3.364 - 5.780), which fits well within the optimal range of 95% of approved drugs (–2.0 to 6.0). As expected, an increased degree of lipophilicity (calculated as LogP_o/w_) was observed to be susceptible to those compounds with long alkyl chains. A close view of the LogP_o/w_ numbers showed that high degrees of lipophilicity within each homologous series appears to correlate strongly with * in-vitro *biological data. This behavior can be explained by considering that high lipophilicity frequently leads to compounds readily taken up into cancer cells in culture, indicating that these compounds can thereafter easily interact with intracellular targets, conducing to more cytotoxic compounds. 

Polar Surface Area (PSA) is the most important physicochemical parameter used to correlate passive molecular transport through membranes and drug-membrane interactions. Predicted PSA numbers (42) for the title compounds showed recommended therapeutic values (ranging from 59.323 to 85.698 Å^2^), suggesting that these smaller compounds could penetrate more efficiently through neoplastic cells. Moreover, the *in-silico *passive transmembrane permeation was calculated for all hybrids using Caco-2 cell monolayers or MDCK cells as a model. Currently, both models are recommended as a simplified *in-vitro *model of intestinal absorption in drug development (43-45). It was found that the synthesized hybrids displayed optimal permeability values (ranging from 454 to 6192 nm/s). 

Finally, we predicted the ability of the hybrids to bind blood plasma proteins, i.e., human serum albumin - HSA (expressed as logK_HSA_), which is the most significant parameter for distribution and transport for anti-cancer formulations in the systemic circulation and play an important role in the early stage of drug discovery. For therapeutic uses, LogK_HSA_ values in the range of -1.5 to 1.5 are recommended for potential drugs (46, 47). The quantitative model suggests that compounds having positive numbers tend to have higher binding affinity to HSA, while negative values could indicate that chemicals show less affinity to HSA binding. Interestingly, all hybrids fit well within the recommended values for 95% of the FDA-approved medications with low positive LogK_HSA_ numbers between 0.162 to 0.833, suggesting that these compounds eventually will be absorbed into the blood, could be rapidly distributed into organs and tissues, would suffer minimal metabolic degradation and not rapid elimination, and in consequence, they would have optimal therapeutic concentrations in plasma to exert some beneficial or protective effects.

*In-silico* physicochemical data, merging *S*-allyl cysteine with sub-units based on anti-inflammatory drugs in a unique structural core provides active compounds with optimal pharmacokinetic properties, making them promissory scaffolds to develop candidates to fight against colorectal cancer.

*Molinspiration bioactivity score calculations*

The pharmacological response for a bioactive compound requires its binding to a specific target, which could provide a potential health benefit. In that context, bioactivity score prediction is currently the most important *in-silico* approach to explore the potential of a compound. This parameter is suitable to predict a drug candidate based on its potential interaction with different human receptors as G protein-coupled receptor (GPCR), ion channel, kinase, nuclear receptor, proteases and enzymes. As a general rule, the most promising drug candidates display larger numbers of bioactivity scores, higher than 0.00, which increases the probability that evaluated compounds would be biologically active. In our study, the novel hybrids based on *S-*allyl cysteine coupled with different molecules FDA-approved for use as anti-inflammatory drugs (salicylic acid, diclofenac, naproxen or ibuprofen) were evaluated via virtual high-throughput screening (vHTS) with the help of Molinspiration chemoinformatic software to demonstrate that the synthesized compounds could be active and thus, could be used to run further biological assays focused on colorectal cancer drug discovery. 

As shown in Table 3, most of the synthesized compounds **9**-**12** were moderately active according to the bioactivity score (from -5.0 to 0.0). Interestingly, hybrids displayed excellent protease inhibition in the range of 0.04 to 0.29, including the most promising compounds **10a-c**, which exhibited bioactivity scores higher than 0.10. In this scenario, since proteolytic enzymes are strongly associated with colorectal cancer development and progression, it is necessary to highlight the importance of designing novel candidates that could inhibit these proteases during cancer chemoprevention (48-50). According to these findings, we can speculate that *in-vitro *activity found for hybrids **10a-c** could be due to inhibition of some proteases associated with cancer, suggesting that molecules based upon molecular hybridization of *S-*allyl cysteine and anti-inflammatory drugs can be considered as a promissory scaffold to discover potential drug candidates to treat colorectal cancer.

*Osiris Drug-Likeness Score and toxicity calculations*

Prediction of drug relevant properties and the overall toxicity of novel hybrids **9**-**12** were carried out using Osiris property explorer, which is a powerful webserver to predict physicochemical and toxicological molecular properties. Calculations were used to estimate the risks of side effects, such as mutagenicity, tumorigenic risk, irritant and reproductive effect, as well as drug-relevant indices that include aqueous solubility (calculated as LogS), drug-likeness and drug score. The toxicity risk predictions locate substructures within a molecule that could enhance the risks of adverse drug reactions on a cell, organ or organism. Notably, toxicity risk assessment revealed that combining *S-*allyl cysteine and NSAIDs fragments in a unique structural unity can lead to synthesized substances with low or null toxicity regarding mutagenicity, tumorigenicity, irritant effect and injury of reproductive system (Green suggests low toxicity risk by Osiris online software).

On the other side, drug-likeness measures based on the OSIRIS web tool were explored based on a drug-likeness model score revealing the potential therapeutic for all hybrids compared to marketed drugs. Osiris database considered that around 15000 nondrug-like chemicals and 3300 FDA-approved drugs have had positive values of the drug-likeness parameter. In contrast, almost all the nondrug like chemicals had negative values (51). A positive number of drug-likeness indicates that the compound is composed mostly of building blocks (fragments) commonly found in marketed drugs. As shown in Table 4, among all compounds evaluated, hybrids **9c, 9d, 10c, 11c, 12a **and** 12c** showed positive drug-likeness values ranging from 0.18 to 3.07 and thus, they could be safe drug candidates, or their structures could be used as models for scaffold-based drug discovery in colorectal cancer chemotherapy. 

In addition, to guarantee safety and optimal drug-likeness indices for the new compounds, data from the Osiris property explorer website indicated that the potential drug score values of compounds **9c** and **12a** were significantly higher (>65%) than the remaining hybrids, which showed values lower than 56%. Finally, from the Osiris property explorer website, we estimated the aqueous solubility value (calculated as LogS) for all the designed compounds. The aqueous solubility is one of the most important biopharmaceutic properties associated with the absorption and distribution of a potential drug. It is, therefore, no surprise that the aqueous solubility plays a key role in the bioavailability of bioactive compounds. In fact, it is well known that more than 95% of the approved drugs can take an ideal range of -6.5 to 0.5 estimated for the LogS parameter. In our case, the LogS values of studied compounds are within the reference range (ranging from -2.42 and -6.30), suggesting their potential as drug-like.

Taken together, the optimal results of safety and drug-likeness together with the acceptable drug score and LogS values estimated for hybrids **9**-**12** would suggest that these compounds have a great chance to advance through the earlier preclinical testing as well as may serve as attractive candidates for future drug development treatments focus on the initiation, progression, and migration in colorectal cancer. 

**Table 1:** Cytotoxic effect of the hybrids against SW480 and CHO-K1 cell lines at 24h and 48h

**Compounds**	**24 (h)**			**48 (h)**		
	**IC**_50_ ** (mM)** **CHO-K1 cells**	**IC**_50_ ** (mM)** **SW480 cells**	**SI**	**IC**_50_ ** (mM)** **CHO-K1 cells**	**IC**_50_ ** (mM)** **SW480 cells**	**SI**
**9a **	0.146 ± 0.016	>0.20	<0.73	0.157 ± 0.005	>0.20	<0.80
**9b **	>0.20	0.160 ± 0.013	>1.25	>0.20	>0.20	>1
**9c **	>0.20	>0.20	>1	>0.20	>0.20	>1
**10a **	0.180 ± 0.012	0.192 ± 0.004	0.93	0.112 ± 0.008	0.120 ± 0.004	0.94
**10b **	>0.2	0.240 ± 0.013	<0.84	>0.20	0.131 ± 0.012****	>1.53
**10c **	0.223 ± 0.005	0.204 ± 0.005	1.09	0.167 ± 0.1	0.158 ± 0.002**	1.06
**11a **	>0.20	>0.20	>1	>0.20	>0.20	>1
**11b **	>0.20	>0.20	>1	>0.20	0.156 ± 0.008**	>1.28
**11c **	>0.20	>0.20	>1	>0.20	0.237 ± 0.012	<0.84
**12a **	>0.20	>0.20	>1	0.201 ± 0.001	0.244 ± 0.011	0.82
**12b**	>0.20	>0.20	>1	0.241 ± 0.018	0.183 ± 0.005	1.31
**12c **	>0.20	0.225 ± 0.01	>1.1	>0.2	0.209 ± 0.005	>1
Aspirin	>0.20	>0.20	>1	>0.20	>0.20	>1
Diclofenac	>0.20	0.267 ±0.010	<0.75	0.202±0.001	0.206 ±0.011	0.98
Ibuprofen	>0.20	>0.20	>1	>0.20	>0.20	>1
Naproxen	>0.20	>0.20	>1	>0.20	>0.20	>1
5-Fluorouracil	0.125 ± 0.022	0.096 ± 0.024	1,29	0.046 ± 0.015	0.037 ± 0.013	1.22

The IC_50_ values were obtained from dose-response curves for each compound. Selectivity index (SI) was calculated by the ratio of IC_50_ values in non-malignant CHO-K1 cells to IC50 of SW480 cells. Data are presented as the mean ± SE of at least three independent experiments. *p-*values lower than 0.05 were considered statistically significant (^**^p < 0.01; ^****^p < 0.0001).

**Tabla 2:** Lipinski’s rule and pharmacokinetic score for the synthesized conjugates **9-12**

**Entry**	**M.W ** ^a^	**PSA** ^b^	**n-Rot Bond**	**n-ON**^c^	**n-OHNH** ^d^	**Log P**_o/w _^e^	**Log K**_HSA_^f^	**App.Caco-2** ^g^	**App.** **MDCK ** **(nm/s)** ^ h^	**HIA** ^i^ **(%)**	**Lipinski Rule of five **
**9a**	295.353	85.698	8	4	2	3.364	0.162	838	509	100	0
**9b**	309.379	83.719	9	4	2	3.746	0.284	854	544	100	0
**9c**	323.406	80.889	10	4	2	3.889	0.334	795	454	100	0
**10a**	453.382	74.958	10	5	2	5.368	0.596	1138	4029	100	0
**10b**	467.409	73.646	11	5	2	5.503	0.637	1259	3463	100	1
**10c**	481.436	73.593	12	5	2	5.780	0.833	1327	6192	100	1
**11a**	387.493	70.654	9	5	1	4.574	0.377	1766	1951	100	0
**11b**	401.520	72.413	10	5	1	4.789	0.476	1647	1472	100	0
**11c**	415.546	66.342	11	5	1	5.388	0.608	2638	2612	100	1
**12a**	363.514	59.323	10	5	1	4.685	0.485	1703	1666	100	0
**12b**	377.541	62.253	11	5	1	5.265	0.658	1872	2152	100	1
**12c**	391.568	59.337	12	5	1	5.493	0.709	2036	2303	100	1

^a^Molecular weight of the hybrid (150-500). ^b^Polar surface area (PSA) (7.0–200 Å^2^). ^c^n-ON number of hydrogen bond acceptors <10. ^d^n-OHNH number of hydrogens bonds donors ≤5. ^e^Octanol/water partition coefficient (LogP_o/w_) (–2.0 to 6.0). ^f^Binding-serum albumin (K_HSA_) (-1.5 to 1.5). ^g^Human intestinal permeation (<25 poor,>500 great). ^h^Madin-Darby canine kidney (MDCK) cells permeation. ^i^Human intestinal absorption (% HIA) (>80% is high, <25% is poor).

**Tabla 3:** Molinspiration bioactivity calculations of the synthesized hybrids **9-12**

**Compounds**	**Bioactivity score** ^a^					
	**GPCR ligand** ^b^	**Ion channel modulator **	**Kinase inhibitor**	**N** **uclear receptor ligand**	**Protease inhibitor**	**Enzyme inhibitor**
**9a**	-0.19	-0.25	-0.67	-0.46	0.04	-0.06
**9b**	-0.20	-0.26	-0.66	-0.41	0.04	-0.10
**9c**	-0.12	-0.24	-0.59	-0.35	0.12	-0.04
**10a**	0.03	-0.11	-0.20	-0.34	0.12	-0.00
**10b**	-0.02	-0.12	-0.23	-0.34	0.10	-0.04
**10c**	0.02	-0.12	-0.22	-0.32	0.12	0.01
**11a**	-0.03	-0.24	-0.55	-0.37	0.16	-0.06
**11b**	-0.08	-0.25	-0.56	-0.39	0.11	-0.10
**11c**	-0.04	-0.24	-0.54	-0.36	0.14	-0.05
**12a**	0.02	-0.20	-0.68	-0.38	0.29	-0.05
**12b**	-0.03	-0.20	-0.69	-0.39	0.25	-0.08
**12c**	0.01	-0.19	-0.65	-0.36	0.27	-0.03

^a^If bioactivity score > 0 (active), −5.0 – 0.0 (moderately active), < −5.0 (inactive): based on activity scores for the six most important FDA-approved drugs ^b^GPCR = G-protein coupled receptor.

**Table 4:** Drug-Likeness Score and toxicity risks calculation of hybrids **9**-**12** based on OSIRIS property explorer

**Compounds**	**Toxicity risk** ^a^				**Drug-relevant properties**		
	**Mutagenicity**	**Tumorigenicity**	**Irritant effect**	**Reproductive effect**	**Solubility ** **(LogS)** ^b^	**Drug-likeness** ^c^	**Drug Score (%)**
**9a**	Green	Green	Green	Green	-2.42	-1.82	52
**9b**	Green	Green	Green	Green	-2.72	-3.01	46
**9c**	Green	Green	Green	Green	-2.99	0.29	69
**10a**	Green	Green	Green	Green	-5.73	0.18	33
**10b**	Green	Green	Green	Green	-6.03	-1.04	24
**10c**	Green	Green	Green	Green	-6.30	2.28	31
**11a**	Green	Green	Green	Green	-4.67	-0.29	46
**11b**	Green	Green	Green	Green	-4.97	-1.4	35
**11c**	Green	Green	Green	Green	-5.24	1.92	48
**12a**	Green	Green	Green	Green	-3.98	0.94	65
**12b**	Green	Green	Green	Green	-4.28	-0.26	47
**12c**	Green	Green	Green	Green	-4.55	3.07	56

^a^Green indicates no risk or low risk; yellow indicates medium risk, and red indicates more toxicity risk. ^b^log S for aqueous solubility (ranging from -6.5 to 0.5 according to 95% of approved Drugs). ^C^A positive number indicates a promising active compound.

## Conclusion

Our results show that hybrids **10b-c**, **11b** and **12b** displayed significant cytotoxic activity against human colon adenocarcinoma cells being significantly more active than parental compounds and exhibiting selectivity comparable to a conventional chemotherapeutic (5-FU), highlighting the potential of the strategy based on molecular hybridization. The SAR analysis showed that the presence of two-carbon atom alkylic chains in the compounds increased the activity, as in hybrids **10b**, **11b** and **12b.**

Calculated pharmacokinetics parameters suggest that merging *S*-allyl cysteine with NSAIDs fragments leads to the formation of compounds with optimal druggability. Besides, the positive bioactivity scores higher than 0.10 against proteases suggest that there could be a possible therapeutic target for the novel hybrids. Moreover, the Osiris Drug Score (>65%) and toxicity risk calculations could indicate that the most active hybrids here evaluated could be used in further studies in the search for different therapeutics alternative against colorectal cancer. 

## Conflict of interest

The authors declare no conflict of interest. 
